# Authenticity Testing and Detection of *Eurycoma longifolia* in Commercial Herbal Products Using Bar-High Resolution Melting Analysis

**DOI:** 10.3390/genes9080408

**Published:** 2018-08-12

**Authors:** Nur Fadhila Fadzil, Alina Wagiran, Faezah Mohd Salleh, Shamsiah Abdullah, Nur Hazwani Mohd Izham

**Affiliations:** 1 Department of Biotechnology & Medical Engineering, Faculty of Biosciences and Medical Engineering, Universiti Teknologi Malaysia, Johor Bahru 81310, Malaysia; fadhila.fadzil@gmail.com (N.F.F.); faezah@fbb.utm.my (F.M.S.); nurizham92@gmail.com (N.H.M.I.); 2 Faculty of Plantation and Agrotechnology, Universiti Teknologi MARA (UiTM), Shah Alam 40450, Selangor, Malaysia; shamsiah3938@salam.uitm.edu.my

**Keywords:** *Eurycoma longifolia*, rbcL, ITS2, authentication, adulteration, Bar-HRM

## Abstract

The present study demonstrated High Resolution Melting (HRM) analysis combined with DNA barcode (Bar-HRM) as a fast and highly sensitive technique for detecting adulterants in *Eurycoma longifolia* commercial herbal products. Targeting the DNA barcoding of the chloroplastic region-ribulose biphosphate carboxylase large chain (rbcL) and the nuclear ribosomal region- internal transcribed spacer 2 (ITS2), PCR amplification and HRM analysis using saturated Eva green dye as the source of fluorescence signals, was accomplished by employing a real-time cycler. The results were further validated by sequencing to identify unknown sequence from Genbank database and to generate phylogenetic tree using neighbour joint (NJ) analysis. Both of the DNA markers exhibited a distinguishable melting temperature and shape of the normalised curve between the reference and the adulterants. In the case of species identification, ITS2 was more successful in differentiating between species. Additionally, detection of admixture sample containing small traces of targeted *E. longifolia* DNA (*w*/*v*) can be detected as low as 5% for rbcL and less than 1% for ITS2, proving the sensitivity and versatility of the HRM analysis. In conclusion, the Bar-HRM analysis is a fast and reliable technique that can effectively detect adulterants in herbal products. Therefore, this will be beneficial for regulatory agencies in order to regulate food safety issues.

## 1. Introduction

According to the World Health Organization, the use of herbal products for treating diseases is practised by 70% of the population in developing countries. As a result, there are large quantities and varieties of raw materials that are sold in markets, and most of them are in processed or modified forms (dried/powdered/infusion); thus, they are impossible to morphologically authenticate using conventional tools. This creates a challenge when trying to accurately distinguish between genuine products and counterfeit ones [1,2]. Targeted species in herbal products may be substituted/adulterated with other unrelated species; poor quality/inferior substances are not only deceitful, but also a threat to consumer health and safety [3]. Hence, the development of an accurate, rapid, and reliable method for species identification and adulterant detection are crucial to ensure the quality and efficiency of the commercial herbal products. 

Malaysia’s forest contains a biodiversity of medicinal plants that have a high potential in the herbal industry. Realising the economic potential of the health value of herbal/medicinal plants, Malaysia’s government declared 11 important herbs, including *Eurycoma longifolia*, to be commercialised to produce high-value products amounting to Ringgit Malaysia 2.2 million in Gross National Income (GNI) by 2020. According to the Malaysian Herbal Corporation, the value of the herbal industry has increased from RM7 million in 2010 and is expected to rise to RM29 billion in 2020 [4]. This indicates that there is a significant demand for herbal products (especially for natural remedies); thus, malpractices such as species substitution are suspected. The significance of marketable herbal products was demonstrated by the increase in product registration percentage (20%) with the National Pharmaceutical Control Bureau (NPCB) to fulfil the rising demand.

*Eurycoma longifolia* is widely acclaimed for its energy-boosting and aphrodisiac properties. It is also known to increase libido effect and it acts as a testosterone booster in males [5,6,7]. The bitterness in Tongkat Ali make it less favorable for direct consumption. Hence, it can usually be found as an additive in health supplements including capsules, tablets and beverages. The crude extract of *E. longifolia* was largely marketed in a mixture of tea, coffee and capsules with some of them being mixed with other herbs [8]. In Malaysia, there were about 200 various *E. longifolia* products distributed all over the country [9]. However, such a claim had stated that *E. longifolia* herbal products in packaging labels may not be true because of the depletion of the raw materials from their natural resources over time [10]. Due to the expensive market price for *E. longifolia* roots (20–25 US dollar/kg) and water extracts (26 USD per 60 capsules), manufacturers may intentionally substitute raw materials with cheaper sources [10,11].

Various criteria and methods of identification have been developed to authenticate medicinal plant species such as morphological analysis, macroscopic and microscopic studies, chemical profiling and simple sequence repeat markers. However, there is currently no best practice for identifying the various plant species in herbal products. The mentioned approaches have several disadvantages such as their accuracy being highly dependent on individual experience/skills, it is difficult to differentiate between closely related substitutes or morphological similarities, they are complex and imprecise, chemical variations among samples exist and impractical practices with various standards are a dilemma. On the other hand, the molecular markers method can detect differences at the DNA level and it offers numerous advantages over the conventional phenotype-based approaches since DNA is stable and detectable in all tissues regardless of the growth environment, development stages or differentiation statuses.

The internal transcribed spacer 2 (ITS2) region has been proposed as a universal barcode for the identification of medicinal plants [12] because of its high discrimination power. Since then, the ITS2 region has been shown to distinguish a wide range of plant species [12,13,14,15]. Other than that, ribulose biphosphate carboxylase large chain (rbcL), was also applied in most land plants due to the ease of amplification ad the suitability for sequencing [16,17]. Because of its universality, more than 10,000 rbcL sequences have been deposited in Genbank to be used as a core DNA barcode in plants [18].

In recent years, novel methods and advancements of molecular techniques, coupled with high resolution melting (HRM) analysis, have allowed for the application of DNA-based approaches. These fast and sensitive techniques characterise nucleic acid samples based on their disassociation behaviour for detecting small sequence differences in PCR-amplified products [19,20]. Several studies have reported the successful application of HRM technology using various DNA barcodes in medicinal plant authentication [15,21,22,23]. Previous publications also claimed that the combination of Bar-HRM analysis is low in cost, simple, highly sensitive and precise in authenticating various plant species and herbal products [24,25,26,27,28]. However, the application of HRM analysis in the authentication of herbal products is still in its initial stages; moreover, the classification and identification of herbal products are limited, especially in developing countries. Hence, this work presents the development of Bar-HRM as a reliable tool for the authentication of herbal plants with focus on ‘Asian Viagra’, *E. longifolia*, and its commercial herbal products.

## 2. Materials and Methods 

### 2.1. Plant Materials, Herbal Products and DNA Isolation

The *E. longifolia* plant was obtained from the Forest Research Institute Malaysia, Kepong, Selangor, Malaysia (FRIM) and was verified by Dr. Richard Chung Cheng Kong with voucher no: (PID 010117-01). Commercial herbal products containing *E. longifolia* as the main ingredient on the packaging label were randomly chosen from Malaysian markets, abbreviated as P1–P6 (Table 1). In addition, five products (P7–P11) which contain *E. longifolia* + *Camellia sinensis* (tea product) and *E. longifolia* + *Coffea* (coffee product) were selected for HRM sensitivity assessment (Table 2).

For DNA isolation, approximately 100 mg of *E. longifolia* root, and 200 mg of the herbal product were separately ground in liquid nitrogen to a fine powder. Then the samples were extracted using NucleoSpin Plant II Kit (Macherey-Nagel, Duren, Germany) as per the manufacturer’s instructions. The yield and purity were assessed by using the Nanodrop 1000 Spectrophotometer (Thermo Fisher Scientific, Waltham, MA, USA) and visualised in 1% (*w*/*v*) agarose gel electrophoresis, viewed by Gel Documentation (Thermo Fisher Scientific-USA). Samples were then diluted to 10 ng/µL working concentration and stored at −20 °C for further use.

### 2.2. Real Time PCR and HRM Analyses

Real time PCR was conducted in 25 µL of the total reaction volume containing 10 µL of 2x Type-it HRM PCR master mix, 15 ng DNA for *E. longifolia*/30 ng DNA for herbal products, 0.2 µM of each primer set and topped up with nano-pure water. The primers used in this work were: rbcLa-Forward: 5′ATGTCACCACAAACAGAGACTAAAGC-3′ [29] and rbcLa-Reverse: 5′-GTAAAATCAAGTCCACCRCG-3′ [30]; as well as ITS2-Forward: 5′-GGG GCG GAT ATT GGC CTC CCC TTG C-3′ and ITS2-Reverse: 5′-GAC GCT TCT CCA GAC TAC AAT-3′ (accession number: KY553292.1 *Eurycoma longifolia* Internal Transcribed Spacer 2). Amplification was then performed by using Rotor Gene Q real time cycler (QIAGEN-Germany).HRM with pre-amplification file was chosen to set up the run. The thermocycling reaction was conducted in 36-well plates starting with a 95 °C initial step for 5 min, followed by 40 cycles of 95 °C for 30 s, 52 °C for 40 s and 72 °C for 30 s. Subsequently, HRM was performed by increasing the temperature at 0.1 °C for each step from 75 °C to 90 °C for rbcL and 80 °C to 95 °C for ITS2. The captured fluorescence signals were then analysed by using the Rotor-Gene Q Series Software version 2.3.1 (QIAGEN-Germany). From the DNA quality assessment, P5–P6 and P10–P11 yielded a very poor DNA quality (Supplementary Materials) and there was no amplification detected (Table 1 and Table 2). Hence, HRM study for these products was not proceeded.

### 2.3. Neighbour Joining Tree Analysis

To corroborate data obtained from the HRM analysis, real time PCR products (*E. longifolia* and P1–P4) were sent for purification and sequencing at 1st Base Malaysia. Sequencing was conducted in both directions with similar primers (Applied Biosystems, Foster City, CA, USA). The sequencing results were first edited by applying the Bioedit Software (version 7.2.6). Data was then checked for high similarity sequences using the basic local Alignment search tool (BLAST). Multiple sequence alignment was then performed using Jalview (version 2.9.0.1.) Finally, the neighbour-joining analysis was constructed using MEGA 6 with 1000 bootstrap replications.

### 2.4. HRM Sensitivity Assessment (Eurycoma longifolia + Camellia sinenesis)

In the HRM sensitivity assessment study of P7–P9, both fresh plant samples *E. longifolia* and *C. sinensis* (tea plant) were used as the genotype references. In order to evaluate the sensitivity and level of detection of adulterants in the admixture samples, *E. longifolia* DNA was mixed with *C. sinensis* DNA (*w*/*v*) in different percentages starting from 0%, 1%, 5%, 10%, 30%, 50% and 75%, in a total amount of 30 ng/µL. The mixture was vortexed slowly to homogenize the sample. This standard served as a reference to detect traces of *E. longifolia* in P7–P9. Lastly, real time PCR amplification and HRM analysis were performed as described above (Section 2.2).

## 3. Results

### 3.1. Bar- HRM Analysis of rbcL and ITS2 for Authentication of Eurycoma Longifolia Herbal Products

**Figure 1:**
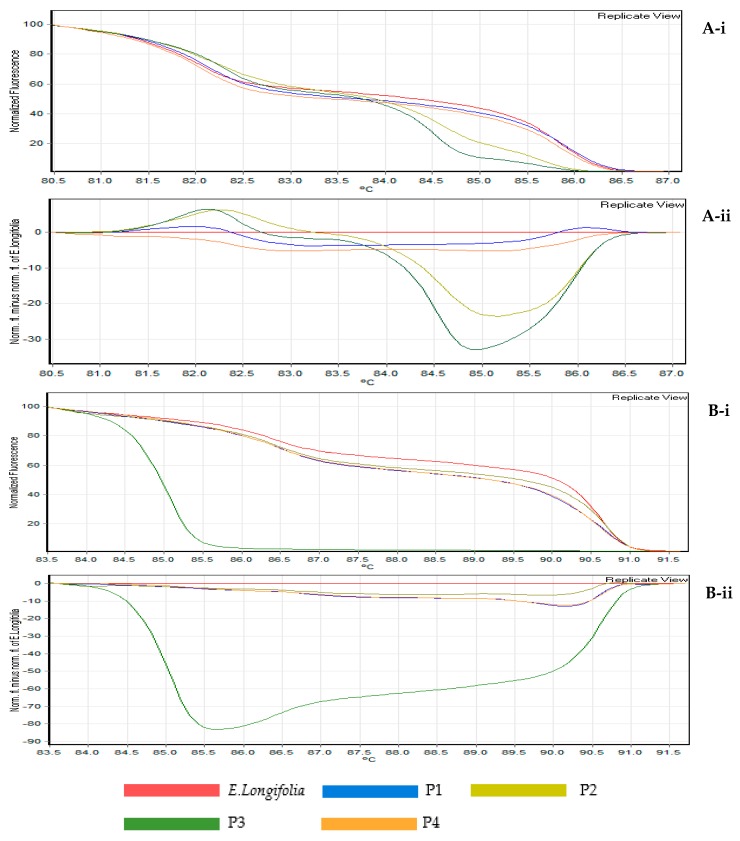
HRM analysis on real time PCR amplicon targeting rbcL (**A-i**) normalised rbcL curves, (**A-ii**) rbcL difference curves; and ITS2 (**B-i**) normalised ITS2 curves, (**B-ii**) ITS2 difference curves.

In the present study, using the shape of melting curves was more informative and time efficient for assessing the differences in targeted species within selective herbal products. The feasibility of the two DNA barcodes of rbcL and ITS2 were compared in this study. The melting curves of HRM were obtained based on the pattern of normalized temperature-shifted curves and the difference plot (Figure 1(A-i,A-ii,B-i,B-ii)). With the aid of the difference curve from the HRM analysis, any samples with close or similar melting temperatures could be differentiated with high resolution and power, compared to the conventional melting curve; by analysing both the melting curve shape and temperature at the same time [19,23]. To further examine and enhance the visualization between the herbal product samples, the difference curve was employed to distinguish subtle dissimilarities by subtracting the fluorescence of each sample with *E. longifolia* root as the reference.

From this experiment, the rbcL region was successfully amplified (approximately 550 bp DNA-fragments) for fresh plant and herbal products (P1–P4). The amplicons were then further analysed with HRM (Figure 1). The results indicated that two products tested (P2 and P3) had shifted normalized melting patterns (Figure 1(A-i)) and they had a high fluorescence difference from the *E. longifolia* baseline reference (Figure 1(A-ii)). These results demonstrated that the two herbal products possibly contained adulterants. For P2, which claimed to contain 100% pure *E. longifolia*, the HRM curve confidently confirmed that this product was adulterated with other species. In the case of P3, the difference in the melting shape curve revealed that this product contained another plant species not indicated in the product label (after sequencing).

**Figure 2:**
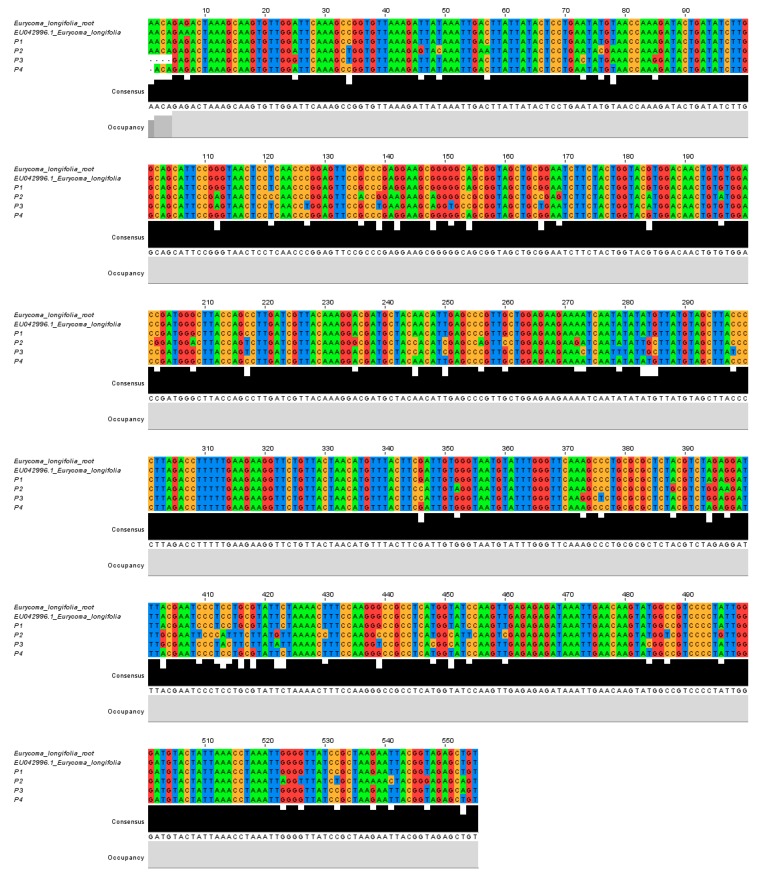
Multiple sequence alignment of rbcL between EU042996.1 *E. longifolia* extracted from NCBI database, *E. longifolia* root and four commercial herbal products: P1, P2, P3 and P4.

**Figure 3:**
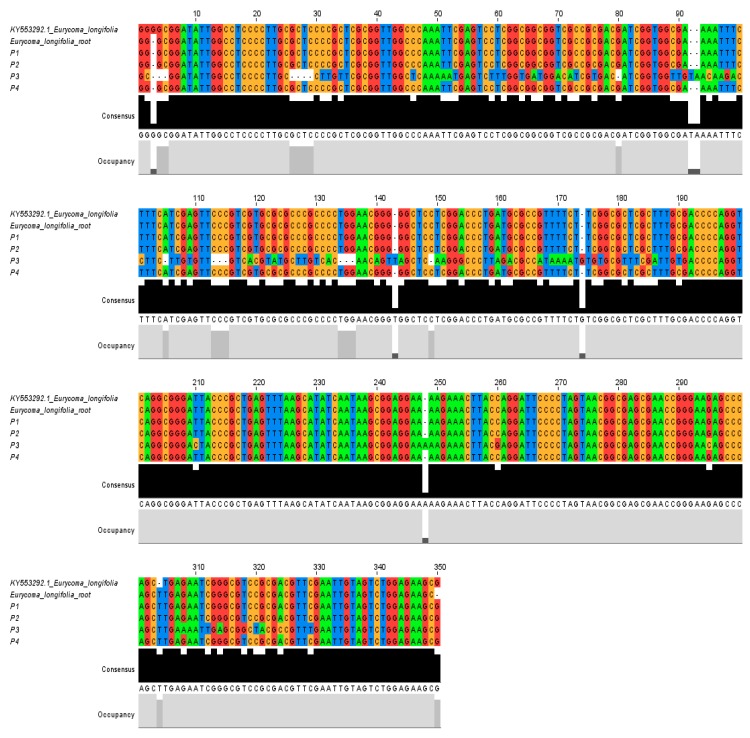
Multiple sequence alignment of ITS2 between KY553292.1 *E. longifolia* extracted from NCBI database, *E. longifolia* root and four commercial herbal products: P1, P2, P3 and P4.

For ITS2, (DNA fragments of approximately 350 bp) was successfully amplified. HRM analysis was be able to detect *E. longifolia* in three herbal products (P1, P2 and P4). As compared to rbcL, the ITS2 difference curve showed a greater fluorescent difference, suggesting that this DNA barcode had higher nucleotide differences; the multiple sequence alignment (Figure 2 and Figure 3) also confirmed the results. This proves that ITS2 is a more precise marker for detecting *E. longifolia* species compared to rbcL.

### 3.2. Bioinformatics Analysis

One of the shortcomings of HRM is that the number and position of mutations within the amplicon cannot be identified by this method alone [20]; hence, real time PCR products were electrophoresed, and sent for purification and sequencing to validate the HRM data. From the multiple sequence alignment, for rbcL (Figure 2), it was confirmed that P2 and P3 showed sequence dissimilarities as compared to *E. longifolia*. For ITS2 (Figure 3), P3 also showed a high sequence variation as compared to the reference plant. These results proves same accordance with the HRM analysis, thus confirming the results. 

**Figure 4:**
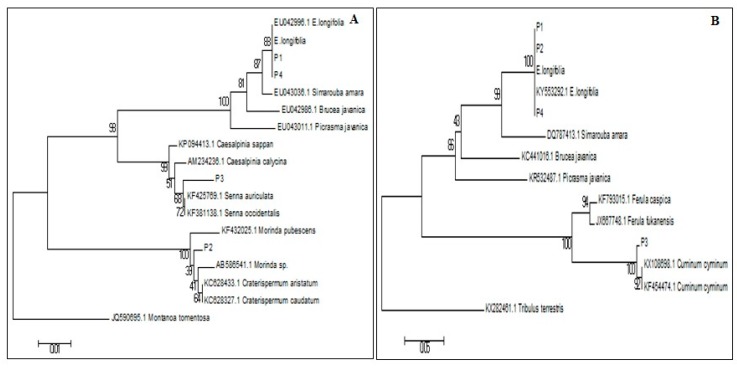
Phylogenetic tree of *E. longifolia* and commercial herbal products (P1, P2, P3, P4) with other plant species constructed from rbcL (**A**) and ITS2 (**B**) sequences using Neighbour Joining (NJ) Analysis with 1000 bootstrap replicates.

The identified sequences were then analysed using neighbour joining (NJ) Analysis. From the rbcL NJ analysis results (Figure 4A), P1 and P4 herbal products were clustered in the same clade as the reference samples; *E. longifolia* root and rbcL *E. longifolia* sequences extracted from NCBI (accession number EU042998.1). For the P2 herbal product, the BLAST result showed a high similarity to *Morinda* and *Craterispermum* genera (accession number AB586541.1; KC628433.1; KC628327.1) from the *Rubiaceae* family; while the P3 herbal product was highly similar to the *Senna* genus from the *Fabaceae* family (accession number KF425769.1; KF381138.1). Plants from these families are not used as aphrodisiacs, but they exhibit other properties in traditional practices.

On the other hand, the NJ analysis of ITS2 (Figure 4B), showed that P1, P2 and P4 herbal products were clustered in the same clade as the *E. longifolia* root and the ITS2 *E. longifolia* retrieved sequence (accession number KY553292.1) with high confidence bootstrap value. The other herbal product, P3, was highly similar to *Cuminum cyminum*, a species from the *Apiaceae* family (accession number KX108698.1; KF454474.1).

Based on the cladogram, it was proven that ITS2 was more suitable for differentiating plants at the species level as compared to rbcL, which can only distinguish up to the genus level. A claim stated that [18], the bioinformatics analysis and sequence alignment shows that rbcL has been able to discriminate plants but only up to some species, and mostly to a congeneric level. On the other hand, in a study of the DNA markers in medicinal plants, ITS2 was proven to exhibit the highest interspecific and intraspecific divergence compared to other DNA markers when distinguishing between species [12].

### 3.3. Detection of Eurycoma longifolia in Admixture Products

**Figure 5:**
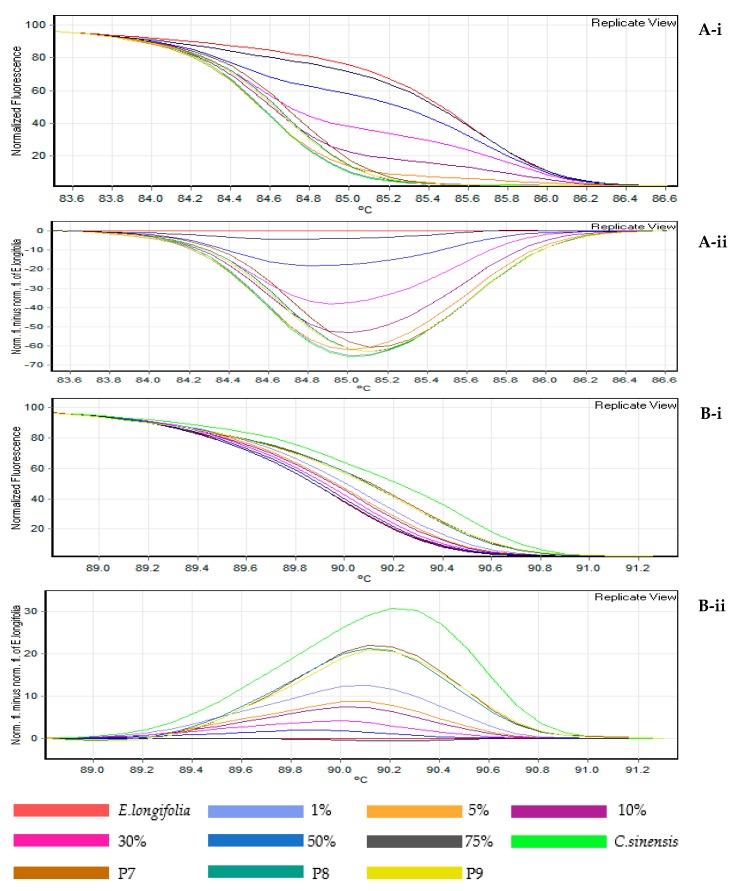
Adulteration of *E. longifolia* mixed in *C. sinensis* to test the sensitivity of HRM analysis in the admixture product. The mixture contained 1%, 5%, 10%, 30%, 50% and 75% *E. longifolia* in *C. sinensis*. HRM analysis targeting rbcL: (**A-i**) normalised rbcL curves, (**A-ii**) rbcL difference curves; and ITS2: (**B-i**) normalised ITS2 curves, (**B-ii**) ITS2 difference curves with *E. longifolia* as a reference, *C. sinensis* and three successfully amplified commercial admixture herbal products: P7, P8 and P9.

To confirm the sensitivity and detection level of the HRM assay by using rbcL and ITS2, this study was evaluated by mixing a series of different percentages of admixtures; *E. longifolia* with *C. sinensis*. As illustrated in Figure 5(A-i,A-ii,B-i,B-ii), the dissociation pattern of rbcL and ITS2 amplicon, respectively, revealed the actual degree of contamination resulting from adulteration at a specific level. This is because, as reported by several studies, amplicon dissociation pattern is influenced and shifted by the quantity of mixed contents in a sample [14,26]. 

The HRM method was able to detect adulterants up to 5% for rbcL and less than 1% for ITS2. This suggests that HRM is a rapid and sensitive method for detecting small amounts of *E. longifolia* species in a mixed sample. Tested herbal products (P7–P9) shows that *E. longifolia* was detected at the baseline limit at 5% using rbcL. This suggest that rbcL does not have enough sequence variations to detect subtle amounts of targeted DNA that are lower than the detection limit. For ITS2, traces of *E. longifolia* (P7–P9) was detectable at less than 1%, showing that ITS2 is more suitable for the detection of *E. longifolia* at a very low concentration.

## 4. Discussion

### An Authentication Method Using Bar-HRM

Developing an accurate and reliable method for the rapid identification of herbal products based on plant species is now possible with HRM. Authentication up to the species and genus levels, is now conceivable based on the melting temperature (Tm) of specific PCR products. However, fraudulent product detection is not easy when the constituent species are in processed form. Normally, DNA extraction from the processed herbal products are highly degraded and with poor DNA quality. Unlike DNA barcoding, Bar-HRM employs sensitive melting curve changes caused from the release of a saturated intercalating dye from DNA duplex denaturation by increasing the temperature [27]. Another advantage of using this method is that the method is performed in a closed-tube system where HRM analysis is directly applied right after amplification in real time cycler, thus reducing carry-over contamination that will influence the positive result. Therefore, the Bar-HRM method proposed in the present study could be applied in cases where DNA barcoding has failed. Previous reports have shown the success of these combined methods for species authentication in medicinal plants or for commercial herbal products [24,25,31,32,33].

DNA that is amplified from mixed herbal products will contribute to a contaminative background that leads to poor sequencing signals, impeding species identification [14,15]; hence, authentication using DNA barcoding alone is impossible. The use of HRM has also been established with the sensitivity detection of adulterants in an admixture sample. For instance, from previous study, the detection of adulterants in medicinal plant admixtures can be assessed at concentrations as low as 1:1000 for unknown species [34]. Another finding has also recorded the detection of targeted DNA as low as 1% in herbal admixture by using HRM analysis [35]. From our study, HRM has proven to be useful in detecting subtle amount of *E. longifolia* depending on the type of DNA region used. The present study has also revealed that even though HRM is proven to be highly sensitive, this method requires the presence of a reference sample of the targeted species. In the case of a sample with more than one species in a product, more references must be included.

## 5. Conclusions

In a summary, the HRM assay proved to be a simple and reliable method for the identification of *E. longifolia* in tested herbal products. The use of ITS2 as a DNA barcode was more reliable than rbcL due to the high discrimination power in both post DNA barcoding and HRM analysis. The DNA barcode coupled with HRM, not only distinguished the fake products from the authentic ones, but also demonstrated the sensitive detection levels of such methods.

## Figures and Tables

**Table 1:** Tested commercial *Eurycoma longifolia* herbal products chosen randomly from the market, abbreviated as P1–P6 (Section 2.1).

Samples	Type of Samples	Labelled Species	Purchased from	rbcL	ITS2
P1	Tea bag	*E. longifolia*	Online	+	+
P2	Tea bag	*E. longifolia*	Online	+	+
P3	Capsule	*E. longifolia* *Cuminum cyminum* *Alpina galangal* *Zingiber officinale*	Herbs store	+	+
P4	Capsule	*E. longifolia*	Online	+	+
P5	Beverage	*E. longifolia*	Herbs store	-	-
P6	Beverage	*E. longifolia*	Herbs store	-	-

**Table 2:** Tested commercial tea bags and coffee instant drink powder containing traces of *E. longiolia* as herbal additives abbreviated as P7–P11 for the High Resolution Melting (HRM) sensitivity assessment (Section 2.4).

Samples	Type of Samples	Labelled Species	Purchased from	rbcL	ITS2
P7	Tea bag	*E. longifolia* *Camellia* *sinensis*	Online	+	+
P8	Tea bag	*E. longifolia* *C. sinensis*	Online	+	+
P9	Tea bag	*E. longifolia* *C. sinensis*	Online	+	+
P10	Instant coffee mix	*E. longifolia* *Coffea*	Herbs store	-	-
P11	Instant coffee mix	*E. longifolia* *Coffea*	Herbs store	-	-

+ Successful amplification; - No amplification.

## Supplementary Materials

The following are available online at http://www.mdpi.com/2073-4425/9/8/408/s1.
