# Supplementary material for: Authenticity Testing and Detection of Eurycoma longifolia in Commercial Herbal Products Using Bar-High Resolution Melting Analysis
Source: Genes (Basel). 2018 Aug 12;9(8):408. doi: 10.3390/genes9080408 (PMC6115996; doi:10.3390/genes9080408)
Supplement: Supplementary file 1 [file genes-09-00408-s001.pdf]

genes

Supplementary Material

Table 1: Samples extracted in this study.

Sample	Absobance ratio 260/280
E.logifolia root	1.77 ± 0.04
P1	1.67 ± 0.02
P2	1.60 ± 0.08
P3	1.72 ± 0.02
P4	1.56 ± 0.04
P5	1.21 ± 0.06
P6	0.79 ± 0.05
C.sinensis leaf	1.83 ± 0.02
P7	1.88 ± 0.01
P8	1.86 ± 0.00
P9	1.82 ± 0.01
Coffea bean	1.61 ± 0.03
P10	0.93 ± 0.23
P11	1.14 ± 0.51

E.longifolia root, P1,P2,P3,P4, *C.sinensis* leaf, P7,P8 and P9 exhibit good DNA quality and were succesfully amplified using both rbcLa and ITS2 primers. However for P5, P6, P10 and P11, they yield a very low DNA quality which hindered PCR amplification and HRM analysis.

Genes **2018**, *9*, x; doi: FOR PEER REVIEW www.mdpi.com/journal/genes

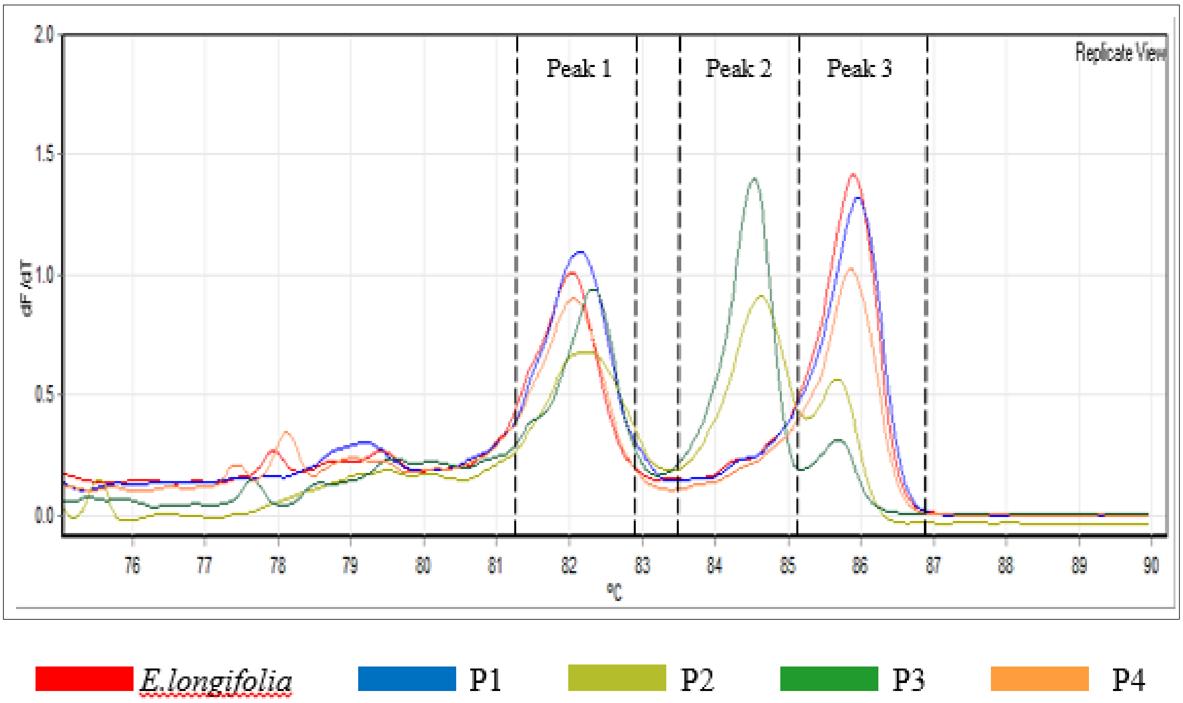

Figure 1: Melt curve peak of *E .longifolia* and four herbal products amplified using rbcLa primer

Table 2: Melting temperature Tm (°C) of *E.longifolia* root and four herbal products amplified using rbcLa primer.

Sample	Peak 1<br>Tm ± SD	Peak 2<br>Tm ± SD	Peak 3<br>Tm ± SD
E.longifolia	82.04 ± 0.02	-	85.90 ± 0.00
P1	82.14 ± 0.06	-	85.97 ± 0.02
P2	82.24 ± 0.26	84.64 ± 0.16	85.69 ± 0.13
P3	82.33 ± 0.04	84.53 ± 0.00	85.69 ± 0.02
P4	82.06 ± 0.04	-	85.87 ± 0.05

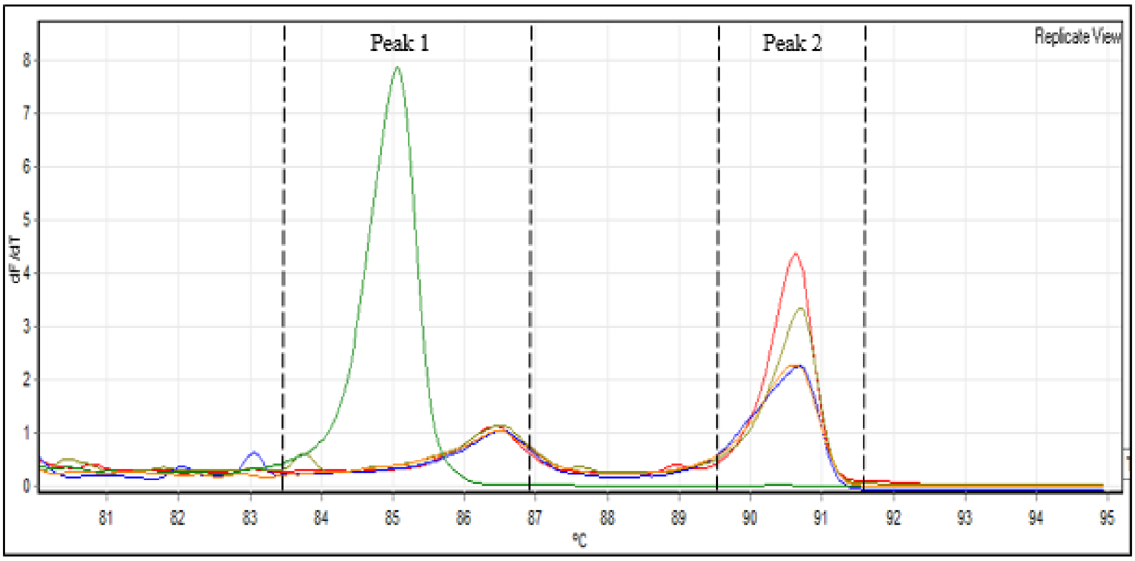

Figure 2: Melt curve peak of *E .longifolia* and four herbal products amplified using rbcLa primer

Table 3: Melting temperature Tm (°C) of *E.longifolia* root and four herbal products amplified using ITS2 primer.

Sample	Peak 1<br>Tm ± SD	Peak 2<br>Tm ± SD
E.longifolia	86.43 ± 0.04	90.62 ± 0.00
P1	86.51 ± 0.01	90.69 ± 0.01
P2	86.50 ± 0.00	90.69 ± 0.01
P3	85.06 ± 0.01	-
P4	86.47 ± 0.09	90.65 ± 0.21
